# Psychopathological symptom network structure in transgender and gender queer youth reporting parental psychological abuse: a network analysis

**DOI:** 10.1186/s12916-021-02091-5

**Published:** 2021-09-22

**Authors:** Yuanyuan Wang, Zhihao Ma, Amanda Wilson, Zhishan Hu, Xin Ying, Meng Han, Zaixu Cui, Runsen Chen

**Affiliations:** 1 Division of Psychology, Faculty of Health and Life Sciences, De Montfort University, Leicester, UK; 2 National Clinical Research Center for Mental Disorders, Department of Psychiatry, and China National Technology Institute on Mental Disorders, The Second Xiangya Hospital of Central South University, Changsha, China; 3 Computational Communication Collaboratory, School of Journalism and Communication, Nanjing University, 163 Xianlin Road, Qixia District, Nanjing, 210023 Jiangsu China; 4 State Key Laboratory of Cognitive Neuroscience and Learning, Beijing Normal University, Beijing, China; 5 Beijing LGBT Center, Beijing, China; 6 Department of Medical Psychology, The School of Health Humanities, Peking University, Beijing, China; 7 Chinese Institute for Brain Research, Beijing, China; 8 Vanke School of Public Health, Tsinghua University, No.30, Shuangqing Road, Haidian District, Beijing, China

**Keywords:** Transgender youth, Family cold violence, Depression, Anxiety, Network analysis

## Abstract

**Background:**

This is the first study to investigate the effect of parental psychological abuse on potential psychopathological symptoms in gender minority youth subgroups, including transgender women, transgender men, and gender queer individuals.

**Methods:**

Data was analysed from the Chinese National Transgender Survey in 2017; the survey was distributed through community-based organizations to transgender adolescents and adults residing in China, with representation from all 32 provinces and autonomous regions. A total of 1293 youth that self-identified as transgender or gender queer completed the study. Measures covered psychopathological symptoms including depression, anxiety, risk of suicideand self-harm. Parental psychological abuse was assessed in terms of neglect and avoidance, force to change, and verbal insults. Both the edges and centralities were computed via network analysis, and the network properties were then compared among the three gender minority subgroups. In addition, linear regression was adopted to test the predictive ability of node centrality for low self-esteem.

**Results:**

Descriptive analysis revealed that among the three subgroups, transgender women had more severe psychopathological symptoms and reported the most psychological abuse. Network analysis revealed that the risk of suicide and self-harm was directly connected with one type of parental psychological abuse (“neglect and avoidance”). Node centrality was significantly associated with the predicting value of the nodes on low self-esteem (*r*^2^ = 0.25, 0.17, 0.31) among all three gender minority subgroups.

**Conclusions:**

The distinctive core psychopathological symptoms, within the networks of the gender minority subgroups, revealed specific symptoms across each group. The significant association between node centrality and low self-esteem indicated the extent of parental psychological abuse. Parental psychological abuse directed towards gender minority youth should be recognized as a form of family cold violence. It is recommended that schools and local communities should support early intervention to improve psychological well-being.

**Supplementary Information:**

The online version contains supplementary material available at 10.1186/s12916-021-02091-5.

## Background

Due to the social stereotype of gender identity being fixed, transgender youth often encounter significant obstacles and distress as they transition from their birth sex to their true gender expression [1]. According to the minority stress theory [2], as transgender youth transition, they experience proximal stressors because their true gender role expression is in contrast to their sex role expectations. The minority stress model further believes that as a result of the proximal stressors transgender individuals can experience considerable psychological distress, which can impair their personal, social, and occupational life [3].

When compared to children’s sexual expression, parents both regulate and discipline children’s gender expression more because gender roles are often expressed openly in public environments [4]. Due to this socialized gender binary norm, parents can struggle with accepting their children’s gender fluidity when their child identifies as transgender and/or gender non-conforming (TGNC) [5]. Parents have reported feelings of grief and loss during the process of accepting a children’s transgender identity [6], and even feeling a “traumatic shock” when children came out to them [7]. A previous study in the United States (US) showed more than 59% of transgender youth initially faced negative reactions from their parents during their gender transition [1]. Moreover, gender non-conforming behaviours displayed by transgender youth were received with severe verbal and physical abuse from parents [1, 8]. It is well documented that a negative parental response is a proximal minority stressor that can elevate the risk of psychiatric disorders, such as anxiety and depression, and is related to the individual’s expectation of rejection [8, 9]. In addition, studies have shown that family rejection is a significant factor predicting suicide attempts and self-harm behaviours among transgender and gender non-conforming adults [10–12].

Considering the detrimental consequence of parental abuse, the objective of this study was to better understand the psychological symptoms that resulted from the abuse received by parents. While there are many studies that have focused on families’ attitudes towards the lesbian, gay, bisexual, and transgender (LGBT) youth community [13–16], the transgender subgroup is underrepresented in this area of research, providing little information to explain the family cold violence experienced. To begin to explore the influence of parental psychological abuse on psychopathology symptoms, the study aimed to investigate the networks of anxiety and depression, the two most frequently reported symptoms, as well as the symptoms of self-harm and suicidal ideations, among transgender youth.

By applying a cooperating net of symptoms, network models can provide insight into the comorbidity with psychiatric disorders [17, 18]. In terms of symptom patterns, network modelling can be used to provide prolific visual and quantitative information by both graphically mapping the connections between symptoms and highlighting the central symptoms [19]. Central symptoms in a network can be defined as the uniquely important and central prognostic indicator(s) [20], with the activation of the central symptoms indicating the spread of impulses within the networks; the spread of impulses can also consequently activate a significant number of additional symptoms [21]. Elliott and colleagues also found that the central symptoms are critical for predicting the treatment outcome and clinical impairment of psychiatric disorders [20]. Thus, for future clinical interventions, targeting the central symptoms of the network could treat disorders more efficiently, instead of aiming to treat all the symptoms simultaneously or as they appear.

Furthermore, previous network analysis research suggests that identifying the central symptoms of a disorder, at baseline, can be used to predict treatment outcomes at the follow-up point(s) [20]. Thus, network analysis can offer insight for clinical interventions by identifying the central symptoms and strengths of mood problems, anxiety, risk of self-harm, and suicide from parental psychological abuse. Network analysis is a novel way to identify unique shared associations in a highly multivariate data set [22]. While previous studies have investigated the impact of abuse on depression and anxiety [23, 24] and the interactive mechanism among the symptoms, the specific trigger for mood disorder symptoms is largely unexplored. By using network analysis, it is possible to assess the specific symptoms triggered by parental psychological abuse, as well as any different symptom causes and actions. More effective interventions could prioritize the central symptoms to prevent a decline in psychiatric patient’s conditions [22]. This study measured parental psychological abuse in terms of verbal insults, purposeful neglect and avoidance of the child’s transgender identity, and forcing the youth to change dressing and appearance. These are the frequently experienced forms of parental psychological abuse towards transgender and gender queer youth, which aims to limit their gender expression and decrease their mental well-being and physical health [1, 25, 26]. Research has shown that the more gender non-conforming the youth is the more likely they would report receiving verbal abuse from parents [1]. Parents in turn reported difficulties in accepting their children’s identity and wanted to challenge their identity [7]. It can be hard for parents of transgender youth to adjust to their children’s change in physical appearance, including dress and behaviours [27], with 60% of parents discouraging youth from dressing contradictory to their birth-assigned sex [28]. Another study showed that parents reported that they attempted to change their child’s gender-atypical behaviours and restrict their gender-atypical dress style [29]. Moreover, parents avoided engaging in conversions about their children’s true gender identity and fluidity [5, 30, 31].

Network analysis has also identified that central symptoms are important predictors for related comorbidities [20]; more specifically, self-esteem was negatively impacted by internalized transphobia in TGNC individuals [32]. Family functioning was found to be associated with the self-esteem of the TGNC youth, better family function improved self-esteem, and consequently, this resulted in better mental health outcomes in terms of depressive symptoms, anxious symptoms, and self-harm [33]. Further research on transgender women also found the co-occurrence of low self-esteem and health problems resulted from social marginalization [34]. Overall, TGNC youth who experienced parental psychological abuse, because of their gender identity, were found to display severe psychopathology symptoms, which led to low self-esteem [34]. Thus, it was hypothesized that central symptoms in the networks could predict low self-esteem in TGNC youth. That is, self-esteem would be associated with the integrated network of depression symptoms, anxiety symptoms, and risk of suicide and self-harm, as a result of parental psychological abuse.

This is the first study to use network analysis to investigate the effect of parental psychological abuse on potential psychopathological symptoms, including anxiety and depression, as well as the risk of self-harm and suicide, among transgender youth. This research aimed to explore these psychopathology symptoms across three subgroups (transgender men, transgender women, and gender queer individuals). It further aimed to identify the specific symptoms within the transgender subgroup by focusing on assessing their network interactions. In addition, the researchers also tested the predictability of the central symptoms in the network for low self-esteem in the transgender and gender queer subgroups. By testing the predictive validity, the study aimed to highlight significant consequences from the central symptoms. This research has implications for educating parents, schools, and communities about the consequences of psychological abuse, and to prevent psychological abuse from spreading through an individual’s network and further affecting the psychological well-being of TGNC youth.

## Methods

### Sampling procedure

A cross-sectional survey was conducted between January and September of 2017. A detailed description of the sampling procedure has been reported elsewhere [12, 35, 36]. In survey-based studies, since the LGBT population is typically a hard-to-reach population [37], all participants were recruited via a two-stage sampling strategy that combined convenience sampling, respondent-driven sampling, and snowball sampling. In the first stage, questionnaires were delivered via LGBT services, educational institutes, social media platforms on public media, and LGBT online communities. In the second stage, participants were invited to share the questionnaires with their TGNC friends and acquaintances. The data set had 5677 survey responses from 32 provinces and autonomous regions. As this study aimed to identify the role of parental psychological abuse on transgender and gender queer youth, the researchers only included participants aged 13 to 29 years old who identified as transgender or queer and in addition had valid responses for depressive symptoms, anxiety symptoms, parental psychological abuse, and risk of suicide and self-harm. The criteria were consistent with a previous study [38]. After exclusion, the data from 1293 cases were included for the secondary data analysis. This study (secondary data analysis) was granted ethical approval by the Ethics Committee at Second Xiangya Hospital, Central South University.

### Survey instruments

All measures for variables analysed in the current study include identification as transgender or gender queer, the Center for Epidemiologic Studies Depression Scale-9 (CESD-9), the Generalized Anxiety Disorder Scale (GAD-7), presence or absence of parental psychological abuse, risk of suicide and self-harm, and the Rosenberg Self-Esteem Scale (RSE), as described in Additional file 1.

### Analytical strategies

In the current study, a series of analytical strategies were adopted. First, a descriptive comparison among the three transgender and queer groups was conducted to provide an overview of the sample. Next, the network analysis of psychopathology symptoms and risk of suicide and self-harm were compared between abused and non-abused groups. Finally, the network analysis was employed to understand network estimation and network comparison and to testify each nodes’ predicting value on self-esteem.

#### Network estimation

The partial correlation networks (without regularization) were based on the total sample of transgender men, transgender women, and gender queer individuals and estimated via the “BGGM” R package, which provides the novel Bayesian methodology to estimate Gaussian graphic models [39]. The stronger partial correlation between two nodes (symptoms or abuses in the current study) indicated a higher likelihood of connection between these two nodes, when controlling for other variables. Based on the results of all correlation coefficients, the strength centrality for each node in different networks was calculated [40].

#### Network comparison

To examine statistical differences among the networks of three different transgender and gender queer groups, the “BGGM” R package to take the pairwise tests was also adopted [39]. The posterior predictive check was used for assessing differences in overall connection strength [41]. Moreover, the posterior distribution for differences of partial correlation coefficients was evaluated. The differences could be inferred if the 95% credible interval excluded zero [42]. To visualize edge differences between the three groups, the edge differences for each pair of groups were plotted as well.

#### Predicting value of nodes

Following current research [20], to test whether the central symptoms with a high centrality had better utility in predicting their associations with self-esteem, Spearman’s rank correlation analysis was used to calculate the associations between each node (including 16 symptoms, 3 forms of psychological abuse, and the node that indicated the risk of suicide and self-harm risk, as well as self-esteem). These associations were interpreted as the predicting value of the nodes. A higher association score revealed a node that had a higher predicting value towards self-esteem. Furthermore, the relationship between predicting value and centrality was demonstrated via a locally weighted scatterplot smoother (LOWESS) estimator [43], which allowed the fitting of nonparametric smoothing curves to scatterplots without prior assumption of curve shapes. Following the LOWESS results, a linear regression analysis was adopted to testify whether nodes with higher centrality had greater predicting value. If values of centrality were significantly related to predicting values, it would confirm that the self-esteem trait was associated with a network integrated of depression, parental psychological abuse, and risk of suicide and self-harm. All the above steps were carried out separately among transgender men, transgender women, and gender queer individuals.

## Results

The mean ages for transgender men, transgender women, and gender queer individuals were 21.93 (SD = 3.84), 21.27 (SD = 3.82), and 20.75 (SD = 3.29), respectively. Most participants had a high education level and lived in the city. More detailed information of participant characteristics can be found in Table 1.

**Table 1:** Participant characteristics

	Transgender men (*N* = 493)	Transgender women (*N* = 441)	Gender queer individuals (*N* = 359)
Age, years: mean (SD)	21.93 (3.84)	21.27 (3.82)	20.75 (3.29)
Education, *N* (%)			
Did not complete high school	22 (4.46)	39 (8.84)	16 (4.46)
Completed high school or equivalent	110 (22.31)	135 (30.61)	91 (25.35)
Associate college and bachelor degree	315 (63.89)	243 (55.10)	235 (65.46)
Master’s degree and above	46 (9.33)	24 (5.44)	17 (4.74)
Residence, *N* (%)			
City	422 (85.60)	364 (82.54)	298 (83.01)
Town and county	53 (10.75)	67 (15.19)	53 (14.76)
Country and others	18 (3.65)	10 (2.27)	8 (2.23)
Employment status, *N* (%)			
Full-time jobs	140 (28.40)	107 (24.26)	66 (18.38)
Part-time jobs	27 (5.48)	27 (6.12)	31 (8.64)
Self-employment	50 (10.14)	51 (11.56)	30 (8.36)
Current not working	26 (5.27)	48 (10.88)	20 (5.57)
Other jobs	37 (7.51)	32 (7.26)	26 (7.24)
Never have a job	213 (43.20)	176 (39.91)	186 (51.81)
Current annual income, *N* (%)			
<¥10,000	282 (57.20)	257 (58.28)	244 (67.97)
¥10,000–24,999	42 (8.52)	53 (12.02)	33 (9.19)
¥25,000–49,999	64 (12.98)	58 (13.15)	35 (9.75)
¥50,000–99,999	64 (12.98)	45 (10.20)	26 (7.24)
≥¥100,000	41 (8.32)	28 (6.35)	21 (5.85)
Current marital or intimate relationship status, *N* (%)			
Single	161 (32.66)	115 (26.08)	113 (31.48)
Unmarried, but with partner (s)	163 (33.06)	106 (24.04)	79 (22.01)
Married	3 (0.61)	6 (1.36)	3 (0.84)
Never have an intimate relationship	166 (33.67)	214 (48.53)	164 (45.68)
Parental education, *N* (%)			
Below primary school	0 (0.00)	4 (0.91)	1 (0.28)
Primary school	24 (4.87)	33 (7.48)	14 (3.90)
Junior high school	84 (17.04)	73 (16.55)	50 (13.93)
Secondary technical school	61 (12.37)	49 (11.11)	32 (8.91)
Senior high school	105 (21.30)	70 (15.87)	61 (16.99)
Associate college	76 (15.42)	77 (17.46)	68 (18.94)
Bachelor degree	111 (22.52)	94 (21.32)	104 (28.97)
Master’s degree and above	32 (6.49)	41 (9.30)	29 (8.08)

The prevalence of the three groups’ parental psychological abuse was slightly over 50% (see Table S2). Specifically, the prevalence of verbal abuse was 49.47% among all participants, the prevalence of neglect and avoidance from parent or guardian to child was 60.25%, and the prevalence of force to change the child back to birth-assigned sex was 63.26%.

In detail, compared with transgender men and gender queer individuals, transgender women reported the worst rates of depression and anxiety. Furthermore, transgender women reported the highest scores on the CESD-9 (mean = 14.71, SD = 7.55), the GAD-7 (mean = 8.71, SD = 5.84), risk of suicide and self-harm (mean= 1.88, SD = 1.40), and the three items of parental psychological abuse (mean_insulting_ = 0.96, SD_insulting_ = 1.21; mean_neglect and avoid_ = 1.35, SD_neglect and avoid_ = 1.30; mean_force to change_ = 1.23, SD_force to change_ = 1.27). The risk for MDD (42.86%) and severe anxiety (17.46%) among transgender women was significantly higher than those for transgender men and gender queer individuals. In addition, the prevalence of verbal abuse (58.05%) and the prevalence of neglect and avoidance from parent or guardian to child (66.67%) were significantly higher for transgender women than for transgender men and gender queer individuals. In addition, transgender women had the lowest score on self-esteem (mean = 2.55, SD = 0.62).

The results of Table 2 reveal that abused participants reported higher depression, anxiety, risk of suicide and self-harm, and low self-esteem. Particularly among transgender men and transgender women, participants with any type of parental psychological abuse reported higher scores on the CESD-9, the GAD-7, and the risk of suicide and self-harm and low scores on self-esteem when compared to those participants who had not experienced parental psychological abuse. However, for gender queer individuals, the scores of the GAD-7 had no significant differences among the abused and non-abused groups. If gender queer individuals suffered "insulting" or "neglect and avoid", they scored higher on CESD-9 and low scores on self-esteem. While if they suffered “force to change” back to the biological sex or not from parents, the results of CESD-9 and self-esteem showing no significant difference among the abused and non-abused groups.

**Table 2:** Comparison of mental health, risk of suicide and self-harm, and self-esteem among abused and non-abused groups

		Insulting								
		Abused group			Non-abused group			Mean Diff.	*T* value	*p* value
		*N*	Mean	SD	*N*	Mean	SD			
All participants	CESD-9	641	14.92	0.29	652	11.59	0.28	3.34	8.29	**<0.001**
	GAD-7	641	8.67	0.23	652	6.80	0.20	1.88	6.19	**<0.001**
	Risk of suicide and self-harm	641	1.73	0.06	652	1.09	0.05	0.64	8.58	**<0.001**
	Self-esteem	641	2.52	0.02	652	2.72	0.02	−0.20	−5.79	**<0.001**
Transgender men	CESD-9	218	13.68	0.52	275	10.50	0.44	3.19	4.70	**<0.001**
	GAD-7	218	8.10	0.39	275	6.19	0.32	1.92	3.83	**<0.001**
	Risk of suicide and self-harm	218	1.69	0.09	275	1.10	0.07	0.59	5.17	**<0.001**
	Self-esteem	218	2.63	0.04	275	2.82	0.04	−0.19	−3.44	**<0.001**
Transgender women	CESD-9	256	16.24	0.47	185	12.59	0.53	3.65	5.15	**<0.001**
	GAD-7	256	9.61	0.39	185	7.46	0.36	2.15	3.87	**<0.001**
	Risk of suicide and self-harm	256	2.17	0.09	185	1.49	0.10	0.68	5.16	**<0.001**
	Self-esteem	256	2.46	0.04	185	2.66	0.04	−0.20	−3.36	**<0.001**
Gender queer individuals	CESD-9	167	14.52	0.51	192	12.18	0.46	2.34	3.40	**0.001**
	GAD-7	167	7.99	0.39	192	7.03	0.33	0.96	1.88	0.061
	Risk of suicide and self-harm	167	1.09	0.10	192	0.69	0.08	0.40	3.14	**0.002**
	Self-esteem	167	2.48	0.04	192	2.63	0.04	−0.15	−2.48	**0.014**
		Neglect and avoid								
		Abused group			Non-abused group			Mean Diff.	*T* value	*p* value
		*N*	Mean	SD	*N*	Mean	SD			
All participants	CESD-9	779	14.33	0.27	514	11.59	0.32	2.73	6.59	**<0.001**
	GAD-7	779	8.39	0.20	514	6.72	0.23	1.67	5.37	**<0.001**
	Risk of suicide and self-harm	779	1.70	1.70	514	0.96	0.96	0.74	9.88	**<0.001**
	Self-esteem	779	2.56	0.02	514	2.72	0.03	−0.15	−4.40	**<0.001**
Transgender men	CESD-9	305	13.02	0.44	188	10.10	0.53	2.93	4.21	**<0.001**
	GAD-7	305	7.79	0.33	188	5.80	0.38	1.99	3.88	**<0.001**
	Risk of suicide and self-harm	305	1.59	0.07	188	0.99	0.09	0.60	5.11	**<0.001**
	Self-esteem	305	2.68	0.04	188	2.83	0.04	−0.15	−2.61	**0.009**
Transgender women	CESD-9	294	15.76	0.43	147	12.61	0.63	3.16	4.21	**<0.001**
	GAD-7	294	9.35	0.34	147	7.42	0.46	1.94	3.32	**0.001**
	Risk of suicide and self-harm	294	2.17	0.08	147	1.31	0.11	0.86	6.35	**<0.001**
	Self-esteem	294	2.49	0.04	147	2.66	0.05	−0.17	−2.65	**0.009**
Gender queer individuals	CESD-9	180	14.19	0.50	179	12.34	0.47	1.85	2.69	**0.008**
	GAD-7	180	7.83	0.35	179	7.12	0.37	0.72	1.41	0.160
	Risk of suicide and self-harm	180	1.12	0.09	179	0.64	0.08	0.48	3.83	**<0.001**
	Self-esteem	180	2.48	0.05	179	2.65	0.04	−0.17	−2.71	**0.007**
		Force to change								
		Abused group			Non-abused group			Mean Diff.	*T* value	*p* value
		*N*	Mean	SD	*N*	Mean	SD			
All participants	CESD-9	818	13.92	0.26	475	12.07	0.34	1.85	4.35	**<0.001**
	GAD-7	818	8.30	0.20	475	6.74	0.24	1.57	4.96	**<0.001**
	Risk of suicide and self-harm	818	1.60	0.05	475	1.07	0.06	0.53	6.85	**<0.001**
	Self-esteem	818	2.58	0.02	475	2.71	0.03	−0.13	−3.74	**<0.001**
Transgender men	CESD-9	322	12.99	0.43	171	9.87	0.54	3.13	4.41	**<0.001**
	GAD-7	322	7.91	0.32	171	5.37	0.38	2.54	4.91	**<0.001**
	Risk of suicide and self-harm	322	1.46	0.07	171	1.18	0.10	0.28	2.32	**0.021**
	Self-esteem	322	2.67	0.04	171	2.86	0.04	−0.19	−3.32	**0.001**
Transgender women	CESD-9	285	15.50	0.44	156	13.26	0.61	2.24	3.00	**0.003**
	GAD-7	285	9.31	0.35	156	7.61	0.44	1.70	2.94	**0.003**
	Risk of suicide and self-harm	285	2.12	0.08	156	1.45	0.11	0.67	4.98	**<0.001**
	Self-esteem	285	2.49	0.04	156	2.64	0.05	−0.15	−2.46	**0.014**
Gender queer individuals	CESD-9	211	13.20	0.45	148	13.36	0.55	−0.16	−0.22	0.822
	GAD-7	211	7.54	0.34	148	7.39	0.39	0.16	0.30	0.765
	Risk of suicide and self-harm	211	1.11	0.09	148	0.55	0.09	0.56	4.44	**<0.001**
	Self-esteem	211	2.54	0.04	148	2.59	0.05	−0.05	−0.85	0.394

Note: Mean Diff. refers to the differences of mean values from two groups

### Network estimation results

**Fig. 1:**
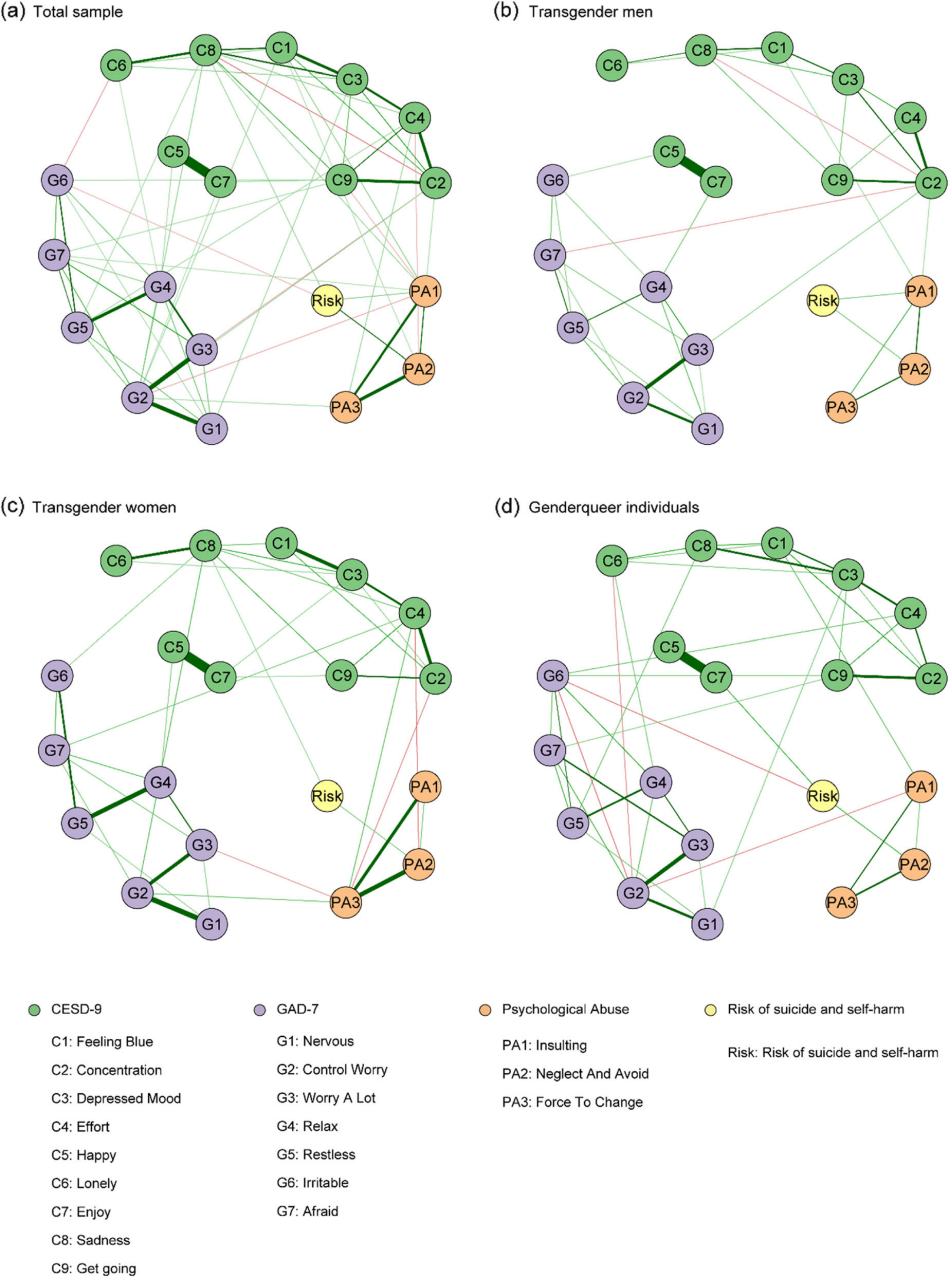
Symptom networks among transgender men, transgender women, and gender queer individuals. The green nodes denote the CESD-9 items, the lavender nodes denote the GAD-7 items, the orange nodes denote the parental psychological abuse, and the yellow node denotes the risk of suicide and self-harm. Meanwhile, the green edges denote the positive correlations and the red edges denote the negative correlations

The estimated networks are displayed in Fig. 1. Detailed edge weights are listed in Tables S3, S4, and S5. First, results revealed several consistent patterns among the three subgroups and that the three types of parental psychological abuse were positively correlated with each other. Among these forms of abuse, “neglect and avoid” was directly linked to the risk of suicide and self-harm, which suggested that suicide and self-harm behaviours were more prevalent among those individuals who were neglected by their parents. Furthermore, the negative association between “effort” and “neglect and avoid” in the transgender women’s network implied that those transgender women with higher “effort” may suffer less “neglect and avoid” from their parents.

Secondly, symptoms significantly linked to “insulting” were different among the three subgroups. For transgender men, two depressive symptoms (“feeling blue” and “concentration”) were directly linked to “insulting”. For transgender women, no symptoms were directly linked to “insulting”, but “effort” and “control worry” could be linked via the node of “force to change”. For gender queer individuals, one depressive symptom (“feeling blue”) was linked to insulting. Moreover, the connection between “control worry” and “insulting” in the gender queer individuals’ network was negatively associated, suggesting “insulting” is the protective factor of “control worry”. Gender queer individuals’ with severe “control worry” may suffer less “insulting” behaviours from their parents.

Third, the “force to change” node was only significantly linked to symptoms in the transgender women network. “Effort” and “control worry” were directly linked to “force to change”, while those transgender women with severe symptoms of “concentration” or “worry a lot” may experience less “force to change”.

Fourth, the risk of suicide and self-harm was linked to depressive symptoms in both the transgender women’s network and gender queer’s networks, whereas it was disconnected with any psychopathological symptoms in the transgender men’s network. The potential connection between any symptom and risk of suicide and self-harm was mediated via “insulting”.

### Network centrality

**Fig. 2:**
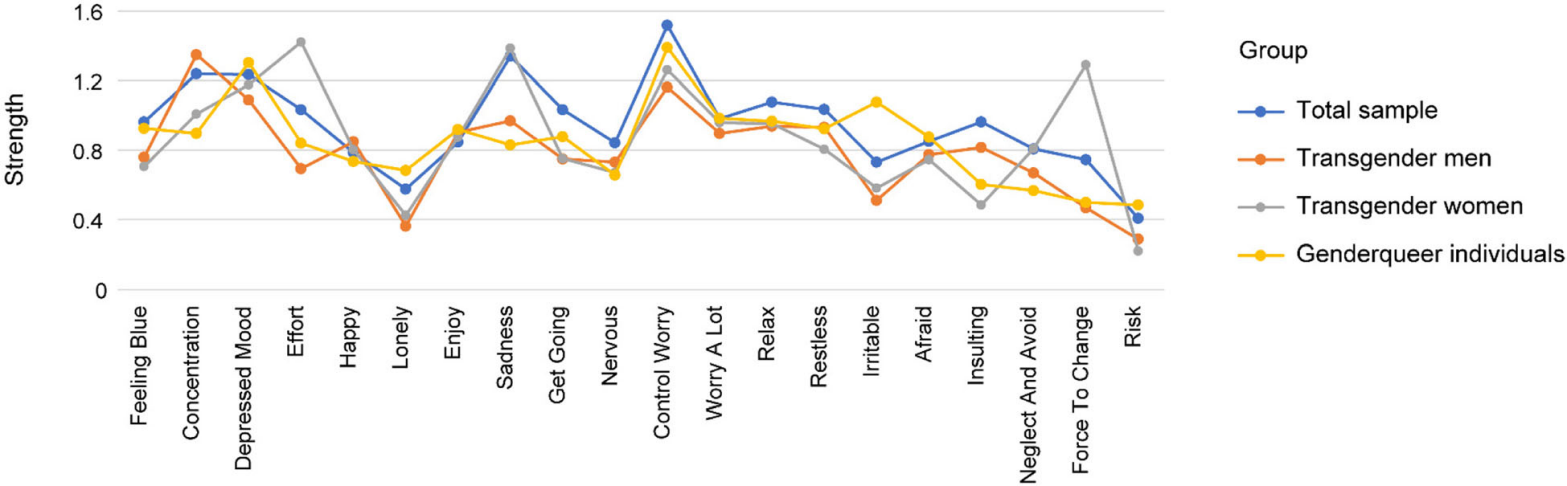
Strength centrality of each node in the networks among transgender men, transgender women, and gender queer individuals. Node strength refers to the number and strength of the direct connections of a node

Figure 2 shows that the strength values of parental psychological abuse were relatively lower than those for most symptoms. However, the strength values of parental psychological abuse among the three groups were not statistically the same. Compared with depressive and anxiety symptoms, the strength values of risk of suicide and self-harm and most abuse nodes were low. However, the strength value of “force to change” in the transgender women’s network was relatively high and implied that the roles of “force to change” for transgender women were significant.

### Network comparison results

**Fig. 3:**
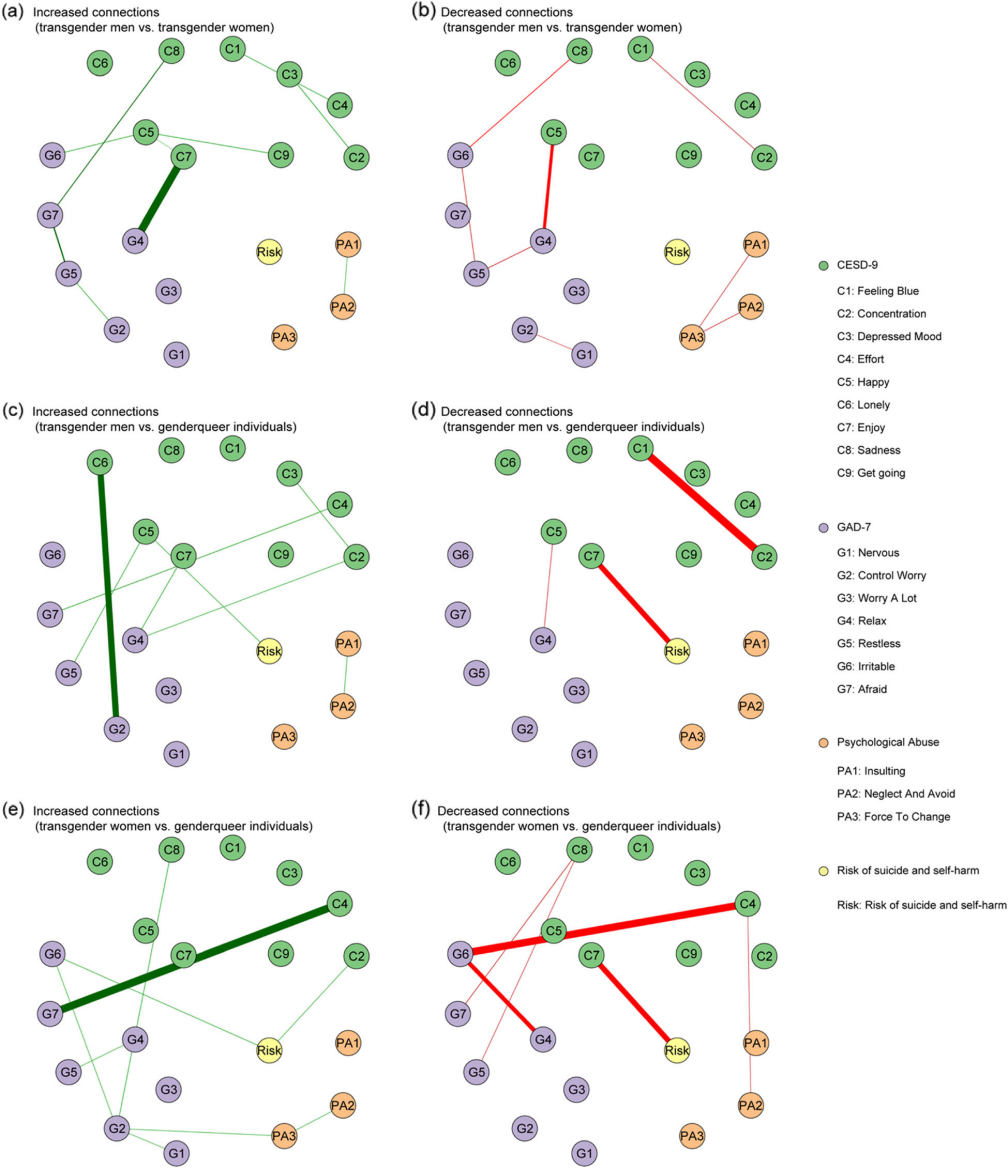
Edges exhibiting significant differences among transgender men, transgender women, and gender queer individuals. The green nodes denote the CESD-9 items, the lavender nodes denote the GAD-9 items, the orange nodes denote the parental psychological abuse, and the yellow nodes denote the risk of suicide and self-harm. **a**, **b** Edge differences between transgender men and transgender women (transgender women as reference). Meanwhile, the green edges denote the increased connections between items at the transgender men network when compared with those in the transgender women network and the red edges denote the decreased ones. **c**, **d** Edge differences between transgender men and gender queer individuals (gender queer individuals as reference). Meanwhile, the green edges denote the increased connections between items in the transgender men’s network when compared with those in the gender queer individuals’ network and the red edges denote the decreased ones. **e**, **f** Edge differences between the transgender women and gender queer individuals (gender queer individuals as reference). Meanwhile, the green edges denote the increased connections between items at the transgender women network when compared with those in the gender queer individuals’ network and the red edges denote the decreased ones. Numbers on edges are the values of the difference

The network differences in edges were compared. No global differences were found among the three groups. However, local differences were found in several edges. Figure 3 reveals significant differences of edge weights among the three groups. Detailed edge differences are listed in Tables S6, S7, and S8. The data concluded that the significant unique pattern of the three groups was as follows.

First, in the transgender men’s network, the two inter-community connections were quite different from transgender women and gender queer individuals’ networks. The connection between “enjoy” and “relax” was stronger while the connection between “happy” and “relax” was significantly weaker, compared with the other two networks.

Second, within the gender queer individuals’ network, one inter-community connection was different from the transgender men’s and transgender women’s networks. The connection between “effort” and “afraid” was significantly weaker compared with the other two networks.

In addition, connections between symptoms and risk of suicide and self-harm were unique in the gender queer individuals’ network. Only the “enjoy” node was significantly connected with “risk”, and the connection was stronger when compared with the other two networks.

### Predicting value of nodes

Spearman’s correlation analysis was used to calculate the predicting value of each node (see Table S9). All correlation values were negative, and a high absolute correlation, indicating that the strength centrality was associated with low self-esteem. Following a recent study’s suggestion [20], the researchers treated the values in Table S9 as predicting values, where higher values indicated that the node was a robust predictor of high self-esteem.

**Fig. 4:**
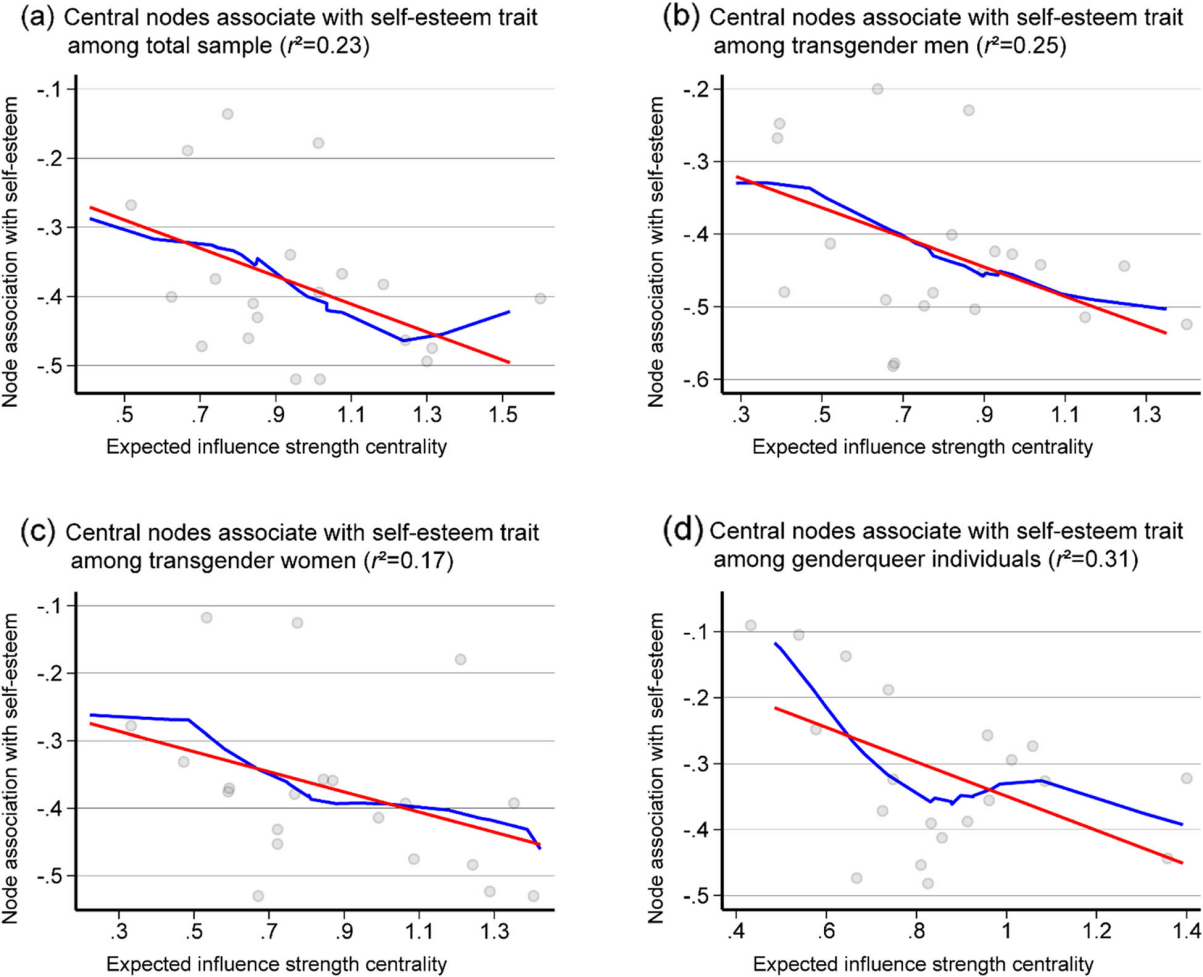
Predicting utility of central nodes on self-esteem trait. Each point denotes a node in the network. A point towards the right on the *x*-axis presents a highly central node. A point towards high on the *y*-axis presents a node that has a high predicting value (highly associated with self-esteem trait). Blue lines denote the estimation results of LOWESS, and red lines denote the estimation results of linear regression

The estimation results of LOWESS in Fig. 4 reveal that the general tendency between the predicting value and strength centrality was negative. Linear regression was utilized to test whether the centrality of nodes was associated with predicting values. Results revealed a significant negative relationships among the total sample (*β* = −0.20, *p* < 0.001), transgender men (*β* = −0.20, *p* < 0.001), transgender women (*β* = −0.15, *p* < 0.05), and gender queer individuals (*β* = −0.26, *p* < 0.05). These results thus verified that low self-esteem was associated with the network integrated symptoms of depression and anxiety, the three forms of parental psychological abuse, and the risk of suicide and self-harm.

## Discussion

This is the first large-scale network analysis on the effect of parental psychological abuse, psychopathological symptoms, and suicide and self-harm among transgender and gender queer adolescents. The results showed a high prevalence of parental psychological abuse among gender minority youth, with slightly over half reporting having experienced parental psychological abuse in one of the three forms. Compared with participants without parental psychological abuse, participants with abuse reported significant symptoms of depression, anxiety, and risk of suicide and self-harm. Within the gender minority groups, the transgender women reported worse rates of depression and anxiety and the highest risk of suicide and self-harm, with more cases of parental psychological abuse reported in all three aspects. The study also identified specific network characteristics in transgender men, transgender women, and gender queer youth. Our results also supported the hypothesis that central symptoms in the network were significantly associated with low self-esteem.

Central symptoms of the network, which integrated depression symptoms, anxiety symptoms, and risk of suicide and self-harm under parental psychological abuse, predicted low self-esteem. This result highlights the impact of the psychopathological symptoms on the transgender and gender queer youth, as it is understood in relation to parental psychological abuse. Low self-esteem was one of the outcomes, which showed the association between negative outcomes of network symptoms. Therefore, it is highly likely there are additional negative outcomes under parental psychological abuse.

The results showed that among the subgroups, transgender women had more psychopathological symptoms. In general, there is a lack of tolerance for gender non-conforming behaviours in boys than girls, and transgender women are more frequently the recipients for verbal and physical abuse [44]. Compared to transgender men, more transgender women reported being physically abused [25]. The research results from this study confirmed the previous findings of the poor conditions experienced by transgender women.

Previous research has shown how parents who were uncomfortable with their child’s gender non-conforming behaviours, and intervened to change their gender back to their birth-assigned sex, did so in order to force their child to meet their societal gender expectations [45]. Previous research also showed that in gender-atypical youth, half of their parents tried to change their behaviours [46]. Similarly, these results revealed that the majority of participants experienced a “force to change” as part of the psychological abuse from their parents or guardians. Furthermore, research reported that children’s gender non-conforming behaviours were negatively associated with parent efforts to “change” their child back to their birth sex [45]. Transgender youth could then internalize parents’ negative attitudes and decrease their self-worth and increase their sensitivity to any non-existing rejection [8]. Gender expression is vital to transgender individuals, since they gain clarity on who they are and experience feelings of happiness through authentic gender identity expression [25].

Family environment and parental support are important for transgender and gender queer youth. Parents have a continuous indisputable important role in the lives of their children, affecting an individual’s sense of self-worth and affecting future relationships [47]. Youth are aware of parents’ discomfort with their gender non-conforming mannerisms and behaviours; as a result, they can start to experience distress and discomfort [25]. Consistently, this study’s findings have demonstrated that youth who experienced parental psychological abuse had significantly worse psychopathology symptoms, in terms of depression and anxiety, as well as an increased risk of suicide and self-harm, than those who did not. Thus, a supportive family environment is vital to the psychological well-being of transgender and gender non-conforming youth. A better family environment is associated with better psychiatric outcomes in transgender and gender non-conforming youth [33]. It is important to understand the specific threats of family rejection to improve minority youth’s psychological well-being [47]. Parents should accept, respect, and appreciate youth from a diverse set of gender expressions [25]. Research suggests that providing interventions to increase parental support for gender non-conforming youth could have a positive effect on well-being [45].

Among the three different types of parental psychological abuse, “insulting” was found to be significantly connected with “feeling blue” and “concentration” in transgender men, connected with “feeling blue” in gender queer individuals, while no symptoms were directly connected with “insulting” in transgender women. That is to say, the common feature of “feeling blue” was connected with “insulting” among transgender men and gender queer individuals, while transgender women displayed no direct symptoms suffered by parental insults. Instead, “force to change” was the only significantly linked symptom in the transgender women’s network. It is likely that “insulting” was indirectly linked to the network via the mediating effect of “force to change”. It is suggested that transgender women suffered increased symptoms from “force to change”, when compared with other subgroups. In Chinese patriarchal culture, son preference is a widely observed social phenomenon, and parents expect to continue the bloodline from their male heir [48]. Previous research of Chinese transgender women showed that their parents tended to blame them for shirking the responsibility of producing offspring as a male heir [49]. This can further be explained by a qualitative study, which revealed that for sons, in particular, parents made conscious efforts to encourage masculinity [50]. Thus, it is likely that parents struggled to accept their born sons’ transition and raised more interference to force them to “change back to normal” to fulfil the male familial responsibility. Consequently, parents were more likely to display intolerance towards their transgender child expressing a feminine appearance. In addition, compared with parents of transgender men, parents of transgender women were more likely to feel that their child needed counselling related to their gender [25]. Though it is important to note this literature is dated and further research is needed to explain why there are differences that occur between the three groups.

Gender queer individuals showed specific characteristics within the network regarding the risk of suicide and self-harm. Compared to transgender men and women, gender queer individuals showed the lowest risk of suicide and self-harm. Compared to transgender women and transgender men, only the “enjoy” node connected with the “risk” node, and the connection was stronger compared with the other two networks in terms of an increased risk for suicide and self-harm. That is, although the risk for suicide and self-harm was relatively low in gender queer individuals, the trigger symptom tended to be clustered and focused on the specific symptom of “enjoy”. Therefore, to reduce suicide and self-harm in gender queer individuals, the current results suggested to primarily pay attention to the “enjoy” related symptom. Moreover, the gender queer group experienced a relatively tolerated parental reaction, with the lowest rates of “neglect and avoid” and “force to change”.

The risk of suicide and self-harm as a result of parental psychological abuse was significantly linked to depressive symptoms in transgender women and gender queer, while there were no direct connections with depressive symptoms in the transgender men network. Compared with other subgroups, the anxiety symptoms were more likely to connect to the risk of suicide and self-harm among transgender women. A previous study also supports gender differences in suicide risks associated with a comorbidity of anxiety and depression, in which the risk was twofold higher in men than women [51]. Consistent with previous finding [51], the current results revealed transgender women had a higher risk of suicide and self-harm than transgender men. The mechanism of gender differences was uncovered, and future studies are needed to investigate the risks of suicide and self-harm associated with mood disorder symptoms among the transgender subgroup [52]. Within the parental abuse, “neglect and avoid” directly linked to the risk of suicide and self-harm in all subgroups, which revealed “neglect and avoid” as the most common symptoms related to parental psychological abuse, that increased suicide and self-harm behaviours in transgender individuals.

Several limitations need to be noted in the interpretation of this research. First, parental psychological abuse was self-reported by transgender youth, and due to the insufficient resources, the actual psychological abuse between parents and youth could not be confirmed. Second, the psychological abuse from the father and mother was not assessed separately. Maternal and paternal reactions towards transgender youth’s gender expression could be different and lead to differences within social network analysis. Future studies should focus on the different delivery of psychological abuse from the father and mother, as well as assessing the different psychopathological symptoms of transgender youth. Third, due to the nature of the cross-sectional design, the causation between parental psychological abuse and mental health problems cannot be confirmed. Future longitudinal studies are needed to investigate the dynamic process and interaction between parental attitudes and mental health status in transgender and gender queer youth. Finally, the current sample was recruited using online purposive sampling, convenience sampling, snowball sampling, and respondent-driven sampling, which are prone to bias but useful for recruiting hard-to-reach populations within any network [53–57]. Considering the limited availability of the resources, the sampling methods adopted were the most applicable and practical options. The sample recruited tended to be younger, from relatively higher socio-economic status families, and have more accessibility to a phone and the Internet. For TGNC, youth from a lower socio-economic status may have less accessibility to the phone and/or Internet and could suffer from higher incidences of parental psychological abuse. It is important for future studies to recruit those who are more disadvantaged in the minority population in order to have a representative view of the diversity within the community.

## Conclusion

In conclusion, this study highlights the high prevalence of parental psychological abuse in transgender and gender queer youth. Network analysis presented distinctive subgroup characteristics with connections between psychological abuse and psychopathological symptoms. Parents of transgender and gender queer youth should support diverse gender expressions and support their child’s transitions from the birth-assigned sex to expressed gender. This study begins to identify parental psychological abuse for transgender youth and indicates some of the various points within network analysis, which should be confirmed in future research, in order to provide further intervention.

## Supplementary Information

**Please delete and do not publish the additional file (Supporting Document12916_2021_2091_DeltaPDF.pdf Supporting Document 12916_2021_2091_DeltaPDF.pdf Additional file 1.** Measures. Tables S1–S9.

## Data Availability

Readers and all interested researchers may contact Runsen Chen (Email address: runsenchen@tsinghua.edu.cn) for details.

## Abbreviations

CESD-9: Center for Epidemiologic Studies Depression Scale-9
GAD-7: Anxiety Disorder Scale
LGBT: Lesbian, gay, bisexual, and transgender
LOWESS: Locally weighted scatterplot smoother
RSE: Rosenberg Self-Esteem Scale
TGNC: Transgender and/or gender non-conforming
US: United States
