# Supplementary material for: Psychopathological symptom network structure in transgender and gender queer youth reporting parental psychological abuse: a network analysis
Source: BMC Med. 2021 Sep 22;19:215. doi: 10.1186/s12916-021-02091-5 (PMC8456702; doi:10.1186/s12916-021-02091-5)
Supplement: Supplementary file 1 — Please delete and do not publish the additional file (Supporting Document12916_2021_2091_DeltaPDF.pdf Supporting Document 12916_2021_2091_DeltaPDF.pdf Additional file 1. Measures. Tables S1–S9. [file 12916_2021_2091_MOESM1_ESM.docx]

***Additional file 1***

***Measures***

***Transgender or gender queer identities***

Consistent with previous studies (Zhu et al., 2019, Chen et al., 2019), participants were asked their assigned biological sex at birth and their self-identified gender. Participants who self-identified as male and were assigned female at birth were coded as transgender men (n=493), participants who self-identified as female and were assigned male at birth were coded as transgender women (n=441), and participants who self-identified as neither male or female were coded as gender queer individuals (n=359). The mean ages of transgender men, transgender women, and gender queer individuals were 21.93 (SD=3.835), 21.27 (SD=3.819), and 20.75 (SD=3.291) respectively. Results of one-way analysis of variance (ANOVA) revealed that the mean ages were significantly different among the three groups (F=10.81, p<0.001). Transgender men were significantly older than transgender women (Mean diff. =0.65, p=0.026) and gender queer individuals (Mean diff. =1.17, p<0.001). The difference in age between transgender women and gender queer individuals was not significant (Mean diff. =0.52, p=0.138).

***Center for Epidemiologic Studies Depression Scale-9 (CESD-9)***

Depression symptoms were assessed via the Center for Epidemiologic Studies Depression Scale-9 (CESD-9), which has been validated within a Chinese population (Jin et al., 2013). The items of CESD-9 and their reference names are listed in Table S1. Participants were asked how many days they had experienced a series of depressive symptoms in the past week. The answers ranged from 0 (less than 1 day) to 3 (5 to 7 days), with a higher score indicating more severe symptoms. The sum score of CESD-9 was used as the measurement of participants’ depression (Cronbach’s alpha=0.910). A score of 10–16 indicates the tendency towards depression, and ≥17 indicates a risk for major depressive disorder (MDD).

***Generalized Anxiety Disorder Scale (GAD-7)***

The Generalized Anxiety Disorder Scale (GAD-7) was used to assess anxiety symptoms (Spitzer et al., 2006), which also has been validated in a Chinese population (Zeng et al., 2013). This instrument consists of 7 items asking participants how many days they have experienced a series of anxiety symptoms (see Table S1) in the past week. The answers range from 0 (less than 1 day) to 3 (5 to 7 days), with higher scores indicating more severe symptoms. The sum score of the GAD-7 was used as the measurement of participants’ anxiety (Cronbach’s alpha=0.928). The guidelines suggests that the cutoff scores of 5, 10, and 15 indicate mild, moderate, and severe anxiety respectively (Spitzer et al., 2006).

***Parental psychological abuse***

Parental psychological abuse was measured by three items, asking participants, how many times they had experienced a series of abusive events (verbal insults, purposeful neglect and avoidance of the child’s transgender identity, and force to change dressing and appearance) from parents or guardians in the past 12 months. The reference names of three items are listed in Table S1. The answer scale ranged from 0 (none) to 3 (above 5 times), with higher scores indicating a more severe extent of abusive behaviors. In addition, these three items were recoded as dichotomous variables to present the prevalence of parental psychological abuse.

***Risk of suicide and self-harm***

In the current study, four items were used to measure the risk of suicide and self-harm: each participant was asked whether they had (1) suicidal ideation, (2) suicide attempts, (3) self-harm ideation, and (4) self-harm attempts as part of the transition to their transgender or gender queer identities. Each item was dichotomous and measured as 0 (no) or 1 (yes). Given an adequate reliability (Cronbach’s alpha=0.759), the sum score of four items was used as the measure of risk of suicide and self-harm (Chen et al., 2014). A higher score indicated a higher risk of suicide or self-harm.

***Rosenberg Self-Esteem Scale (RSE)***

The Rosenberg Self-Esteem Scale (RSE) was used to assess participants’ self-esteem (Rosenberg, 2015), which has been validated in a Chinese population (Wu et al., 2017). This scale consists of 10 items evaluating feelings about one’s self in both positive and negative statements. The answers range from 1 (not at all) to 4 (always). After reversing all negative statements, the mean score of the RSE was used as the measurement of participants’ global self-esteem (Cronbach’s alpha=0.913). Higher values indicated a greater sense of global self-esteem.

Table S1 Questionnaires and Items Used for the Assessment of Psychopathology Symptoms, Psychological Abuse, and Risk of Suicide and Self-harm.

|  | Reference Name | Item |
| --- | --- | --- |
| CESD-9 | Feeling Blue | I felt that I could not shake off the blues even with help from my family or friend |
|  | Concentration | I had trouble keeping my mind on what I was doing |
|  | Depressed Mood | I felt depressed |
|  | Effort | I felt that everything I did was an effort |
|  | Happy* | I was happy |
|  | Lonely | I felt lonely |
|  | Enjoy* | I enjoyed life |
|  | Sadness | I felt sad |
|  | Get going | I could not get going |
| GAD-7 | Nervous | Feeling nervous, anxious or on edge |
|  | Control Worry | Not being able to stop or control worrying |
|  | Worry A Lot | Worrying too much about different things |
|  | Relax | Trouble relaxing |
|  | Restless | Being so restless that it is hard to sit still |
|  | Irritable | Becoming easily annoyed or irritable |
|  | Afraid | Feeling afraid as if something awful might happen |
| Psychological abuse | Insulting | Verbal insulting |
|  | Neglect And Avoid | Purposeful neglect and avoid child being transgender |
|  | Force To Change | Force to change dressing and appearance |
| Risk of suicide and self-harm | Risk | Suicidal ideation |
|  |  | Suicide attempts |
|  |  | Self-harm ideation |
|  |  | Self-harm attempts |

*Reversed items.

Table S2 Comparison of Parental Psychological Abuse, Risk of suicide and self-harm, Mental health, and Self-esteem among Three Groups: Transgender men, Transgender women, and Gender queer individuals.

|  | Total sample | | Transgender men | | Transgender women | | Gender queer individuals | | F value | P value |
| --- | --- | --- | --- | --- | --- | --- | --- | --- | --- | --- |
|  | Mean | SD | Mean | SD | Mean | SD | Mean | SD |  |  |
| CESD-9 | 13.24 | 7.42 | 11.91 | 7.63 | 14.71 | 7.55 | 13.26 | 6.59 | 17.03 | **<0.001** |
| GAD-7 | 7.73 | 5.52 | 7.03 | 5.59 | 8.71 | 5.84 | 7.48 | 4.83 | 11.36 | **<0.001** |
| Risk of suicide and self-harm | 1.41 | 1.37 | 1.36 | 1.30 | 1.88 | 1.40 | 0.88 | 1.21 | 58.92 | **<0.001** |
| Insulting | 0.69 | 1.08 | 0.56 | 1.00 | 0.96 | 1.21 | 0.55 | 0.94 | 20.84 | **<0.001** |
| Neglect and avoid | 0.69 | 1.24 | 1.05 | 1.24 | 1.35 | 1.30 | 0.71 | 1.07 | 27.37 | **<0.001** |
| Force to change | 1.02 | 1.19 | 0.99 | 1.17 | 1.23 | 1.27 | 0.80 | 1.07 | 13.45 | **<0.001** |
| Self-esteem | 2.62 | 0.62 | 2.74 | 0.62 | 2.55 | 0.62 | 2.56 | 0.59 | 13.66 | **<0.001** |
| Prevalence |  |  |  |  |  |  |  |  |  |  |
| Tendency to depression | 29.78% | | 28.60% | | 29.25% | | 32.03% | | 0.63 | 0.534 |
| Major Depressive Disorder | 34.96% | | 28.19% | | 42.86% | | 34.54% | | 11.19 | **<0.001** |
| Mild anxiety | 39.06% | | 37.53% | | 38.10% | | 42.34% | | 1.14 | 0.320 |
| Moderate anxiety | 17.71% | | 13.79% | | 20.18% | | 20.06% | | 4.21 | **0.015** |
| Severe anxiety | 12.76% | | 11.36% | | 17.46% | | 8.91% | | 7.26 | **0.001** |
| Insulting | 49.57% | | 44.22% | | 58.05% | | 46.52% | | 9.96 | **<0.001** |
| Neglect and avoid | 60.25% | | 61.87% | | 66.67% | | 50.14% | | 11.91 | **<0.001** |
| Force to change | 63.26% | | 65.31% | | 64.63% | | 58.77% | | 2.18 | 0.113 |

Table S3 Estimated Edge Weights of the Transgender Men’s Network.

|  | Feeling Blue | Concentration | Depressed Mood | Effort | Happy | Lonely | Enjoy | Sadness | Get Going | Nervous | Control Worry | Worry A Lot | Relax | Restless | Irritable | Afraid | Insulting | Neglect And Avoid | Force To Change | Risk |
| --- | --- | --- | --- | --- | --- | --- | --- | --- | --- | --- | --- | --- | --- | --- | --- | --- | --- | --- | --- | --- |
| Feeling Blue | NA | NA | 0.27 | NA | NA | 0.11 | NA | 0.28 | NA | NA | NA | NA | NA | NA | NA | NA | 0.10 | NA | NA | NA |
| Concentration | NA | NA | 0.27 | 0.35 | NA | NA | NA | -0.10 | 0.32 | NA | 0.12 | NA | NA | NA | NA | -0.10 | 0.10 | NA | NA | NA |
| Depressed Mood | 0.27 | 0.27 | NA | 0.22 | NA | NA | NA | 0.19 | 0.15 | NA | NA | NA | NA | NA | NA | NA | NA | NA | NA | NA |
| Effort | NA | 0.35 | 0.22 | NA | NA | NA | NA | NA | 0.13 | NA | NA | NA | NA | NA | NA | NA | NA | NA | NA | NA |
| Happy | NA | NA | NA | NA | NA | NA | 0.76 | NA | NA | NA | NA | NA | NA | NA | 0.09 | NA | NA | NA | NA | NA |
| Lonely | 0.11 | NA | NA | NA | NA | NA | NA | 0.25 | NA | NA | NA | NA | NA | NA | NA | NA | NA | NA | NA | NA |
| Enjoy | NA | NA | NA | NA | 0.76 | NA | NA | NA | NA | NA | NA | NA | 0.15 | NA | NA | NA | NA | NA | NA | NA |
| Sadness | 0.28 | -0.10 | 0.19 | NA | NA | 0.25 | NA | NA | 0.15 | NA | NA | NA | NA | NA | NA | NA | NA | NA | NA | NA |
| Get Going | NA | 0.32 | 0.15 | 0.13 | NA | NA | NA | 0.15 | NA | NA | NA | NA | NA | NA | NA | NA | NA | NA | NA | NA |
| Nervous | NA | NA | NA | NA | NA | NA | NA | NA | NA | NA | 0.34 | 0.10 | 0.18 | 0.12 | NA | NA | NA | NA | NA | NA |
| Control Worry | NA | 0.12 | NA | NA | NA | NA | NA | NA | NA | 0.34 | NA | 0.42 | NA | 0.18 | NA | 0.11 | NA | NA | NA | NA |
| Worry A Lot | NA | NA | NA | NA | NA | NA | NA | NA | NA | 0.10 | 0.42 | NA | 0.25 | NA | NA | 0.14 | NA | NA | NA | NA |
| Relax | NA | NA | NA | NA | NA | NA | 0.15 | NA | NA | 0.18 | NA | 0.25 | NA | 0.26 | 0.11 | NA | NA | NA | NA | NA |
| Restless | NA | NA | NA | NA | NA | NA | NA | NA | NA | 0.12 | 0.18 | NA | 0.26 | NA | 0.13 | 0.25 | NA | NA | NA | NA |
| Irritable | NA | NA | NA | NA | 0.09 | NA | NA | NA | NA | NA | NA | NA | 0.11 | 0.13 | NA | 0.18 | NA | NA | NA | NA |
| Afraid | NA | -0.10 | NA | NA | NA | NA | NA | NA | NA | NA | 0.11 | 0.14 | NA | 0.25 | 0.18 | NA | NA | NA | NA | NA |
| Insulting | 0.10 | 0.10 | NA | NA | NA | NA | NA | NA | NA | NA | NA | NA | NA | NA | NA | NA | NA | 0.27 | 0.21 | 0.15 |
| Neglect And Avoid | NA | NA | NA | NA | NA | NA | NA | NA | NA | NA | NA | NA | NA | NA | NA | NA | 0.27 | NA | 0.26 | 0.14 |
| Force To Change | NA | NA | NA | NA | NA | NA | NA | NA | NA | NA | NA | NA | NA | NA | NA | NA | 0.21 | 0.26 | NA | NA |
| Risk | NA | NA | NA | NA | NA | NA | NA | NA | NA | NA | NA | NA | NA | NA | NA | NA | 0.15 | 0.14 | NA | NA |

Table S4 Estimated Edge Weights of the Transgender Women’s Network.

|  | Feeling Blue | Concentration | Depressed Mood | Effort | Happy | Lonely | Enjoy | Sadness | Get Going | Nervous | Control Worry | Worry A Lot | Relax | Restless | Irritable | Afraid | Insulting | Neglect And Avoid | Force To Change | Risk |
| --- | --- | --- | --- | --- | --- | --- | --- | --- | --- | --- | --- | --- | --- | --- | --- | --- | --- | --- | --- | --- |
| Feeling Blue | NA | 0.18 | 0.37 | NA | NA | NA | NA | 0.16 | NA | NA | NA | NA | NA | NA | NA | NA | NA | NA | NA | NA |
| Concentration | 0.18 | NA | 0.10 | 0.35 | NA | NA | NA | NA | 0.26 | NA | NA | NA | NA | NA | NA | NA | NA | NA | -0.12 | NA |
| Depressed Mood | 0.37 | 0.10 | NA | 0.29 | NA | 0.10 | 0.10 | 0.21 | NA | NA | NA | NA | NA | NA | NA | NA | NA | NA | NA | NA |
| Effort | NA | 0.35 | 0.29 | NA | NA | NA | NA | 0.14 | 0.21 | NA | NA | NA | NA | NA | NA | 0.13 | NA | -0.14 | 0.16 | NA |
| Happy | NA | NA | NA | NA | NA | NA | 0.68 | NA | NA | NA | NA | NA | 0.13 | NA | NA | NA | NA | NA | NA | NA |
| Lonely | NA | NA | 0.10 | NA | NA | NA | NA | 0.32 | NA | NA | NA | NA | NA | NA | NA | NA | NA | NA | NA | NA |
| Enjoy | NA | NA | 0.10 | NA | 0.68 | NA | NA | NA | 0.10 | NA | NA | NA | NA | NA | NA | NA | NA | NA | NA | NA |
| Sadness | 0.16 | NA | 0.21 | 0.14 | NA | 0.32 | NA | NA | 0.18 | NA | 0.17 | NA | NA | NA | 0.11 | NA | NA | NA | NA | 0.10 |
| Get Going | NA | 0.26 | NA | 0.21 | NA | NA | 0.10 | 0.18 | NA | NA | NA | NA | NA | NA | NA | NA | NA | NA | NA | NA |
| Nervous | NA | NA | NA | NA | NA | NA | NA | NA | NA | NA | 0.45 | 0.12 | NA | 0.11 | NA | NA | NA | NA | NA | NA |
| Control Worry | NA | NA | NA | NA | NA | NA | NA | 0.17 | NA | 0.45 | NA | 0.36 | NA | NA | NA | 0.13 | NA | NA | 0.15 | NA |
| Worry A Lot | NA | NA | NA | NA | NA | NA | NA | NA | NA | 0.12 | 0.36 | NA | 0.25 | NA | NA | 0.13 | NA | NA | -0.10 | NA |
| Relax | NA | NA | NA | NA | 0.13 | NA | NA | NA | NA | NA | NA | 0.25 | NA | 0.41 | NA | 0.17 | NA | NA | NA | NA |
| Restless | NA | NA | NA | NA | NA | NA | NA | NA | NA | 0.11 | NA | NA | 0.41 | NA | 0.29 | NA | NA | NA | NA | NA |
| Irritable | NA | NA | NA | NA | NA | NA | NA | 0.11 | NA | NA | NA | NA | NA | 0.29 | NA | 0.19 | NA | NA | NA | NA |
| Afraid | NA | NA | NA | 0.13 | NA | NA | NA | NA | NA | NA | 0.13 | 0.13 | 0.17 | NA | 0.19 | NA | NA | NA | NA | NA |
| Insulting | NA | NA | NA | NA | NA | NA | NA | NA | NA | NA | NA | NA | NA | NA | NA | NA | NA | 0.14 | 0.34 | NA |
| Neglect And Avoid | NA | NA | NA | -0.14 | NA | NA | NA | NA | NA | NA | NA | NA | NA | NA | NA | NA | 0.14 | NA | 0.41 | 0.13 |
| Force To Change | NA | -0.12 | NA | 0.16 | NA | NA | NA | NA | NA | NA | 0.15 | -0.10 | NA | NA | NA | NA | 0.34 | 0.41 | NA | NA |
| Risk | NA | NA | NA | NA | NA | NA | NA | 0.10 | NA | NA | NA | NA | NA | NA | NA | NA | NA | 0.13 | NA | NA |

Table S5 Estimated Edge Weights of the Gender Queer’s Network.

|  | Feeling Blue | Concentration | Depressed Mood | Effort | Happy | Lonely | Enjoy | Sadness | Get Going | Nervous | Control Worry | Worry A Lot | Relax | Restless | Irritable | Afraid | Insulting | Neglect And Avoid | Force To Change | Risk |
| --- | --- | --- | --- | --- | --- | --- | --- | --- | --- | --- | --- | --- | --- | --- | --- | --- | --- | --- | --- | --- |
| Feeling Blue | NA | 0.21 | 0.25 | NA | NA | 0.12 | NA | 0.21 | NA | NA | NA | NA | NA | NA | NA | NA | 0.14 | NA | NA | NA |
| Concentration | 0.21 | 0.00 | 0.12 | 0.24 | NA | NA | NA | NA | 0.33 | NA | NA | NA | NA | NA | NA | NA | NA | NA | NA | NA |
| Depressed Mood | 0.25 | 0.12 | 0.00 | 0.28 | NA | 0.13 | NA | 0.28 | 0.14 | 0.11 | NA | NA | NA | NA | NA | NA | NA | NA | NA | NA |
| Effort | NA | 0.24 | 0.28 | 0.00 | NA | NA | NA | NA | 0.19 | NA | NA | NA | NA | NA | 0.14 | NA | NA | NA | NA | NA |
| Happy | NA | NA | NA | NA | 0.00 | NA | 0.74 | NA | NA | NA | NA | NA | NA | NA | NA | NA | NA | NA | NA | NA |
| Lonely | 0.12 | NA | 0.13 | NA | NA | 0.00 | NA | NA | NA | NA | -0.11 | NA | 0.12 | NA | NA | NA | NA | NA | NA | NA |
| Enjoy | NA | NA | NA | NA | 0.74 | NA | 0.00 | NA | NA | NA | NA | NA | NA | NA | NA | NA | NA | NA | NA | 0.18 |
| Sadness | 0.21 | NA | 0.28 | NA | NA | 0.21 | NA | 0.00 | NA | NA | NA | NA | NA | 0.13 | NA | NA | NA | NA | NA | NA |
| Get Going | NA | 0.33 | 0.14 | 0.19 | NA | NA | NA | NA | 0.00 | NA | NA | NA | NA | NA | 0.12 | 0.11 | NA | NA | NA | NA |
| Nervous | NA | NA | 0.11 | NA | NA | NA | NA | NA | NA | NA | 0.32 | 0.11 | NA | 0.12 | NA | NA | NA | NA | NA | NA |
| Control Worry | NA | NA | NA | NA | NA | -0.11 | NA | NA | NA | 0.32 | 0.00 | 0.40 | 0.15 | NA | -0.13 | 0.18 | -0.11 | NA | NA | NA |
| Worry A Lot | NA | NA | NA | NA | NA | NA | NA | NA | NA | 0.11 | 0.40 | 0.00 | 0.22 | NA | NA | 0.25 | NA | NA | NA | NA |
| Relax | NA | NA | NA | NA | NA | 0.12 | NA | NA | NA | NA | 0.15 | 0.22 | NA | 0.28 | 0.20 | NA | NA | NA | NA | NA |
| Restless | NA | NA | NA | NA | NA | NA | NA | 0.13 | NA | 0.12 | NA | NA | 0.28 | NA | 0.21 | 0.18 | NA | NA | NA | NA |
| Irritable | NA | NA | NA | 0.14 | NA | NA | NA | NA | 0.12 | NA | -0.13 | NA | 0.20 | 0.21 | NA | 0.15 | NA | NA | NA | -0.13 |
| Afraid | NA | NA | NA | NA | NA | NA | NA | NA | 0.11 | NA | 0.18 | 0.25 | NA | 0.18 | 0.15 | NA | NA | NA | NA | NA |
| Insulting | 0.14 | NA | NA | NA | NA | NA | NA | NA | NA | NA | -0.11 | NA | NA | NA | NA | NA | 0.00 | 0.12 | 0.23 | NA |
| Neglect And Avoid | NA | NA | NA | NA | NA | NA | NA | NA | NA | NA | NA | NA | NA | NA | NA | NA | 0.12 | 0.00 | 0.27 | 0.18 |
| Force To Change | NA | NA | NA | NA | NA | NA | NA | NA | NA | NA | NA | NA | NA | NA | NA | NA | 0.23 | 0.27 | 0.00 | NA |
| Risk | NA | NA | NA | NA | NA | NA | 0.18 | NA | NA | NA | NA | NA | NA | NA | -0.13 | NA | NA | 0.18 | NA | NA |

Table S6 Edge Differences between the Transgender Men’s Network and the Transgender Women’s Network (Ref. = transgender women network).

|  | Feeling Blue | Concentration | Depressed Mood | Effort | Happy | Lonely | Enjoy | Sadness | Get Going | Nervous | Control Worry | Worry A Lot | Relax | Restless | Irritable | Afraid | Insulting | Neglect And Avoid | Force To Change | Risk |
| --- | --- | --- | --- | --- | --- | --- | --- | --- | --- | --- | --- | --- | --- | --- | --- | --- | --- | --- | --- | --- |
| Feeling Blue | NA | -0.14 | NA | 0.14 | NA | NA | NA | NA | NA | NA | NA | NA | NA | NA | NA | NA | NA | NA | NA | NA |
| Concentration | -0.14 | NA | 0.16 | NA | NA | NA | NA | NA | NA | NA | NA | NA | NA | NA | NA | NA | NA | NA | NA | NA |
| Depressed Mood | NA | 0.16 | NA | NA | NA | NA | NA | NA | NA | NA | NA | NA | NA | NA | NA | NA | NA | NA | NA | NA |
| Effort | 0.14 | NA | NA | NA | NA | NA | NA | NA | NA | NA | NA | NA | NA | NA | NA | NA | NA | NA | NA | NA |
| Happy | NA | NA | NA | NA | NA | NA | 0.08 | NA | 0.16 | NA | NA | NA | -0.18 | NA | 0.15 | NA | NA | NA | NA | NA |
| Lonely | NA | NA | NA | NA | NA | NA | NA | NA | NA | NA | NA | NA | NA | NA | NA | NA | NA | NA | NA | NA |
| Enjoy | NA | NA | NA | NA | 0.08 | NA | NA | NA | NA | NA | NA | NA | 0.20 | NA | NA | NA | NA | NA | NA | NA |
| Sadness | NA | NA | NA | NA | NA | NA | NA | NA | NA | NA | NA | NA | NA | NA | -0.16 | 0.16 | NA | NA | NA | NA |
| Get Going | NA | NA | NA | NA | 0.16 | NA | NA | NA | NA | NA | NA | NA | NA | NA | NA | NA | NA | NA | NA | NA |
| Nervous | NA | NA | NA | NA | NA | NA | NA | NA | NA | NA | -0.11 | NA | NA | NA | NA | NA | NA | NA | NA | NA |
| Control Worry | NA | NA | NA | NA | NA | NA | NA | NA | NA | -0.11 | NA | NA | NA | 0.14 | NA | NA | NA | NA | NA | NA |
| Worry A Lot | NA | NA | NA | NA | NA | NA | NA | NA | NA | NA | NA | NA | NA | NA | NA | NA | NA | NA | NA | NA |
| Relax | NA | NA | NA | NA | -0.18 | NA | 0.20 | NA | NA | NA | NA | NA | NA | -0.16 | NA | NA | NA | NA | NA | NA |
| Restless | NA | NA | NA | NA | NA | NA | NA | NA | NA | NA | 0.14 | NA | -0.16 | NA | -0.16 | 0.17 | NA | NA | NA | NA |
| Irritable | NA | NA | NA | NA | 0.15 | NA | NA | -0.16 | NA | NA | NA | NA | NA | -0.16 | NA | NA | NA | NA | NA | NA |
| Afraid | NA | NA | NA | NA | NA | NA | NA | 0.16 | NA | NA | NA | NA | NA | 0.17 | NA | NA | NA | NA | NA | NA |
| Insulting | NA | NA | NA | NA | NA | NA | NA | NA | NA | NA | NA | NA | NA | NA | NA | NA | NA | 0.13 | -0.14 | NA |
| Neglect And Avoid | NA | NA | NA | NA | NA | NA | NA | NA | NA | NA | NA | NA | NA | NA | NA | NA | 0.13 | NA | -0.15 | NA |
| Force To Change | NA | NA | NA | NA | NA | NA | NA | NA | NA | NA | NA | NA | NA | NA | NA | NA | -0.14 | -0.15 | NA | NA |
| Risk | NA | NA | NA | NA | NA | NA | NA | NA | NA | NA | NA | NA | NA | NA | NA | NA | NA | NA | NA | NA |

Table S7 Edge Differences between the Transgender Men’s Network and the Gender Queer’s Network (Ref. = gender queer network).

|  | Feeling Blue | Concentration | Depressed Mood | Effort | Happy | Lonely | Enjoy | Sadness | Get Going | Nervous | Control Worry | Worry A Lot | Relax | Restless | Irritable | Afraid | Insulting | Neglect And Avoid | Force To Change | Risk |
| --- | --- | --- | --- | --- | --- | --- | --- | --- | --- | --- | --- | --- | --- | --- | --- | --- | --- | --- | --- | --- |
| Feeling Blue | NA | -0.17 | NA | NA | NA | NA | NA | NA | NA | NA | NA | NA | NA | NA | NA | NA | NA | NA | NA | NA |
| Concentration | -0.17 | NA | 0.15 | NA | NA | NA | NA | NA | NA | NA | NA | NA | 0.14 | NA | NA | NA | NA | NA | NA | NA |
| Depressed Mood | NA | 0.15 | NA | NA | NA | NA | NA | NA | NA | NA | NA | NA | NA | NA | NA | NA | NA | NA | NA | NA |
| Effort | NA | NA | NA | NA | NA | NA | NA | NA | NA | NA | NA | NA | NA | NA | NA | 0.16 | NA | NA | NA | NA |
| Happy | NA | NA | NA | NA | NA | NA | NA | NA | NA | NA | NA | NA | -0.15 | 0.15 | NA | NA | NA | NA | NA | 0.14 |
| Lonely | NA | NA | NA | NA | NA | NA | NA | NA | NA | NA | 0.17 | NA | NA | NA | NA | NA | NA | NA | NA | NA |
| Enjoy | NA | NA | NA | NA | NA | NA | NA | NA | NA | NA | NA | NA | 0.15 | NA | NA | NA | NA | NA | NA | -0.17 |
| Sadness | NA | NA | NA | NA | NA | NA | NA | NA | NA | NA | NA | NA | NA | NA | NA | NA | NA | NA | NA | NA |
| Get Going | NA | NA | NA | NA | NA | NA | NA | NA | NA | NA | NA | NA | NA | NA | NA | NA | NA | NA | NA | NA |
| Nervous | NA | NA | NA | NA | NA | NA | NA | NA | NA | NA | NA | NA | NA | NA | NA | NA | NA | NA | NA | NA |
| Control Worry | NA | NA | NA | NA | NA | 0.17 | NA | NA | NA | NA | NA | NA | NA | NA | NA | NA | NA | NA | NA | NA |
| Worry A Lot | NA | NA | NA | NA | NA | NA | NA | NA | NA | NA | NA | NA | NA | NA | NA | NA | NA | NA | NA | NA |
| Relax | NA | 0.14 | NA | NA | -0.15 | NA | 0.15 | NA | NA | NA | NA | NA | NA | NA | NA | NA | NA | NA | NA | NA |
| Restless | NA | NA | NA | NA | 0.15 | NA | NA | NA | NA | NA | NA | NA | NA | NA | NA | NA | NA | NA | NA | NA |
| Irritable | NA | NA | NA | NA | NA | NA | NA | NA | NA | NA | NA | NA | NA | NA | NA | NA | NA | NA | NA | NA |
| Afraid | NA | NA | NA | 0.16 | NA | NA | NA | NA | NA | NA | NA | NA | NA | NA | NA | NA | NA | NA | NA | NA |
| Insulting | NA | NA | NA | NA | NA | NA | NA | NA | NA | NA | NA | NA | NA | NA | NA | NA | NA | 0.14 | NA | NA |
| Neglect And Avoid | NA | NA | NA | NA | NA | NA | NA | NA | NA | NA | NA | NA | NA | NA | NA | NA | 0.14 | NA | NA | NA |
| Force To Change | NA | NA | NA | NA | NA | NA | NA | NA | NA | NA | NA | NA | NA | NA | NA | NA | NA | NA | NA | NA |
| Risk | NA | NA | NA | NA | 0.14 | NA | -0.17 | NA | NA | NA | NA | NA | NA | NA | NA | NA | NA | NA | NA | NA |

Table S8 Edge Differences between the Transgender Women’s Network and Gender Queer’s Network (Ref. = gender queer network).

|  | Feeling Blue | Concentration | Depressed Mood | Effort | Happy | Lonely | Enjoy | Sadness | Get Going | Nervous | Control Worry | Worry A Lot | Relax | Restless | Irritable | Afraid | Insulting | Neglect And Avoid | Force To Change | Risk |
| --- | --- | --- | --- | --- | --- | --- | --- | --- | --- | --- | --- | --- | --- | --- | --- | --- | --- | --- | --- | --- |
| Feeling Blue | NA | NA | NA | NA | NA | NA | NA | NA | NA | NA | NA | NA | NA | NA | NA | NA | NA | NA | NA | NA |
| Concentration | NA | NA | NA | NA | NA | NA | NA | NA | NA | NA | NA | NA | NA | NA | NA | NA | NA | NA | NA | 0.14 |
| Depressed Mood | NA | NA | NA | NA | NA | NA | NA | NA | NA | NA | NA | NA | NA | NA | NA | NA | NA | NA | NA | NA |
| Effort | NA | NA | NA | NA | NA | NA | NA | NA | NA | NA | NA | NA | NA | NA | -0.21 | 0.21 | NA | -0.15 | NA | NA |
| Happy | NA | NA | NA | NA | NA | NA | NA | NA | NA | NA | NA | NA | NA | NA | NA | NA | NA | NA | NA | NA |
| Lonely | NA | NA | NA | NA | NA | NA | NA | NA | NA | NA | NA | NA | NA | NA | NA | NA | NA | NA | NA | NA |
| Enjoy | NA | NA | NA | NA | NA | NA | NA | NA | NA | NA | NA | NA | NA | NA | NA | NA | NA | NA | NA | -0.20 |
| Sadness | NA | NA | NA | NA | NA | NA | NA | NA | NA | NA | 0.17 | NA | NA | -0.15 | NA | -0.17 | NA | NA | NA | NA |
| Get Going | NA | NA | NA | NA | NA | NA | NA | NA | NA | NA | NA | NA | NA | NA | NA | NA | NA | NA | NA | NA |
| Nervous | NA | NA | NA | NA | NA | NA | NA | NA | NA | NA | 0.14 | NA | NA | NA | NA | NA | NA | NA | NA | NA |
| Control Worry | NA | NA | NA | NA | NA | NA | NA | 0.17 | NA | 0.14 | NA | NA | NA | NA | 0.15 | NA | NA | NA | 0.15 | NA |
| Worry A Lot | NA | NA | NA | NA | NA | NA | NA | NA | NA | NA | NA | NA | NA | NA | NA | NA | NA | NA | NA | NA |
| Relax | NA | NA | NA | NA | NA | NA | NA | NA | NA | NA | NA | NA | NA | 0.14 | -0.20 | NA | NA | NA | NA | NA |
| Restless | NA | NA | NA | NA | NA | NA | NA | -0.15 | NA | NA | NA | NA | 0.14 | NA | NA | NA | NA | NA | NA | NA |
| Irritable | NA | NA | NA | -0.21 | NA | NA | NA | NA | NA | NA | 0.15 | NA | -0.20 | NA | NA | NA | NA | NA | NA | 0.16 |
| Afraid | NA | NA | NA | 0.21 | NA | NA | NA | -0.17 | NA | NA | NA | NA | NA | NA | NA | NA | NA | NA | NA | NA |
| Insulting | NA | NA | NA | NA | NA | NA | NA | NA | NA | NA | NA | NA | NA | NA | NA | NA | NA | NA | NA | NA |
| Neglect And Avoid | NA | NA | NA | -0.15 | NA | NA | NA | NA | NA | NA | NA | NA | NA | NA | NA | NA | NA | NA | 0.14 | NA |
| Force To Change | NA | NA | NA | NA | NA | NA | NA | NA | NA | NA | 0.15 | NA | NA | NA | NA | NA | NA | 0.14 | NA | NA |
| Risk | NA | 0.14 | NA | NA | NA | NA | -0.20 | NA | NA | NA | NA | NA | NA | NA | 0.16 | NA | NA | NA | NA | NA |

Table S9 Predicting value of each nodes.

| Nodes | Total sample | Transgender men | Transgender women | Genderqueer individuals |
| --- | --- | --- | --- | --- |
| Feeling Blue | -0.43 | -0.49 | -0.37 | -0.39 |
| Concentration | -0.46 | -0.51 | -0.46 | -0.34 |
| Depressed Mood | -0.50 | -0.52 | -0.49 | -0.45 |
| Effort | -0.51 | -0.54 | -0.50 | -0.44 |
| Happy | -0.44 | -0.45 | -0.42 | -0.44 |
| Lonely | -0.38 | -0.46 | -0.31 | -0.35 |
| Enjoy | -0.46 | -0.50 | -0.41 | -0.44 |
| Sadness | -0.46 | -0.50 | -0.52 | -0.32 |
| Get Going | -0.52 | -0.56 | -0.53 | -0.43 |
| Nervous | -0.37 | -0.43 | -0.41 | -0.22 |
| Control Worry | -0.42 | -0.46 | -0.41 | -0.34 |
| Worry A Lot | -0.39 | -0.42 | -0.41 | -0.29 |
| Relax | -0.37 | -0.43 | -0.38 | -0.26 |
| Restless | -0.37 | -0.43 | -0.36 | -0.26 |
| Irritable | -0.34 | -0.38 | -0.34 | -0.29 |
| Afraid | -0.42 | -0.46 | -0.39 | -0.37 |
| Insulting | -0.20 | -0.25 | -0.14 | -0.16 |
| Neglect And Avoid | -0.16 | -0.21 | -0.15 | -0.13 |
| Force To Change | -0.16 | -0.22 | -0.15 | -0.06 |
| Risk | -0.26 | -0.26 | -0.27 | -0.24 |

Predicting value was determined by computing Spearman’s correlations between each node and self-esteem.
